#  The California 2020 Medi-Cal Expansion to Young Adults and Coverage Among Noncitizens

**DOI:** 10.1001/jamanetworkopen.2026.12332

**Published:** 2026-05-13

**Authors:** Russell Leonard, Brandy J. Lipton

**Affiliations:** 1 Department of Economics, University of California, Irvine; 2 Department of Health, Society, and Behavior, University of California, Irvine; 3 Center for Health Economics & Policy Studies, San Diego State University, San Diego, California

## Abstract

**Importance:**

Undocumented immigrants are more than 5 times as likely as US citizens to be uninsured. Before 2020, undocumented young adults aged 19 to 25 years in California were eligible for restricted-scope Medi-Cal, which only covers emergency services.

**Objective:**

To examine the association of the California 2020 full-scope Medi-Cal expansion to young adults aged 19 to 25 years regardless of immigration status with coverage outcomes and to assess subgroup differences by race and ethnicity, sex, and age.

**Design, Setting, and Participants:**

This cross sectional study included American Community Survey respondents who were noncitizens aged 19 to 25 years before (2016-2019) and after (2021-2022) the policy’s implementation in California; the treatment group was compared with California noncitizens aged 26 to 32 years and young adults aged 19 to 25 and 26 to 32 years from 6 comparison states (Arizona, Florida, Illinois, Nevada, New York, and Texas). Analysis was conducted from January 2024 to August 2025.

**Exposure:**

California’s 2020 Medi-Cal expansion.

**Main Outcomes and Measures:**

Triple difference analysis was used to estimate the association of the California Medi-Cal expansion with health insurance coverage (any, Medicaid, and private coverage) among noncitizens aged 19 to 25 years relative to California noncitizens aged 26 to 32 years and young adults in the 6 comparison states.

**Results:**

The sample included 19 773 and 32 515 noncitizen American Community Survey respondents in California aged 19 to 25 years and 26 to 32 years, respectively, and 28 535 and 43 213 individuals aged 19 to 25 years and 26 to 32 years, respectively, residing in comparison states. Baseline weighted percentages for the 19- to 25-year treatment group included 52.1% (95% CI, 51.0%-53.2%) male, 31.9% (95% CI, 30.7%-33.0%) Asian non-Hispanic, 1.8% (95% CI, 1.5%-2.2%) Black non-Hispanic, 54.6% (95% CI, 53.4%-55.9%) Hispanic, 9.7% (95% CI, 8.9%-10.5%) White non-Hispanic, and 2.0% (95% CI, 1.6%-2.3%) other race non-Hispanic. Medi-Cal expansion was associated with a 4.2 (95% CI, 1.3-7.1)–percentage-point increase in Medicaid and a 3.5 (95% CI, 0.2-6.8)–percentage-point increase in any coverage. In subgroup analyses, percentage-point increases in Medicaid were statistically significant for Hispanic young adults (6.7 [95% CI, 2.6-10.9] percentage points), males (3.6 [95% CI, 0.1-7.1] percentage points), females (5.0 [95% CI, 0.7-9.3] percentage points), those aged 19 to 22 years (4.4 [95% CI, 0.7-8.1] percentage points), and those aged 23 to 25 years (4.0 [95% CI, 0.7-7.3] percentage points). In post hoc analyses, the estimates translated to increases in Medi-Cal and any coverage of 24.4 and 20.3 percentage points, or 30 665 and 25 554 young adults, respectively.

**Conclusions and Relevance:**

In this cross-sectional study, the California 2020 Medi-Cal expansion was associated with significant coverage gains. Because the American Community Survey did not distinguish between restricted- and full-scope Medi-Cal, the analysis may have underestimated coverage increases, and further research is warranted to understand the health care and economic costs and benefits of California’s expansion.

## Key Points

**Question:**

What was the association of the California 2020 Medi-Cal expansion to young adults regardless of immigration status with the uninsured rate and coverage among noncitizens aged 19 to 25 years?

**Findings:**

This cross-sectional study of 19 773 California noncitizens aged 19 to 25 years found that the 2020 Medi-Cal expansion was associated with statistically significant increases in Medi-Cal coverage (4.2 percentage points) and any coverage of (3.5 percentage points), with no significant change in private insurance.

**Meaning:**

These findings suggest that expansion was associated with significant gains in Medi-Cal coverage and any coverage.

## Teaser

This cross-sectional study examines the 2020 California Medi-Cal expansion to young adults aged 19 to 25 years and explores associations of the expansion with health care coverage among young adult noncitizens.

## Introduction

Undocumented immigrants are more than 5 times as likely as US citizens to be uninsured.^1^ Restrictions on eligibility for Medicaid, Medicare, and subsidized Marketplace plans by citizenship, together with lower access to employer-sponsored insurance, likely explain much of this gap. Coverage gaps by citizenship contribute to health care disparities by race and ethnicity since most undocumented people are Latino or Asian.^2,3^

While Medicaid eligibility depends on immigration status in most states, 15 states including the District of Columbia have extended state-funded coverage to children regardless of documentation status, and 8 states have done so for some groups of adults.^4^ California expanded Medi-Cal to undocumented young adults up to 26 years of age on January 1, 2020. Prior to 2020, this group was only eligible for restricted-scope Medi-Cal, which covers a limited range of services such as emergency department visits.^5^ Full-scope coverage is far more comprehensive, covering primary care, mental health, and dental care, among other services.^5^ Noncitizens with a qualifying immigration status (eg, lawful permanent residents, asylees, refugees, and pregnant noncitizens) were already eligible for full-scope Medi-Cal before 2020.^6^

This study assessed the association of California’s 2020 expansion with the uninsured rate and coverage sources among noncitizen young adults using a triple difference approach and 2016 to 2022 data from the American Community Survey (ACS). Research on state expansions to undocumented pregnant people indicates increases in use of prenatal care and improvements in birth outcomes.^7,8^ Analogously, studies suggest that state expansions to undocumented children increase health insurance coverage and improve preventative health care access.^9,10,11,12^

Fewer studies leverage the timing of coverage expansions to nonpregnant adults to examine these effects. Our analysis provides timely evidence to contribute to ongoing state and federal policy debates on extending coverage to immigrant populations.

## Methods

This cross-sectional study was considered to be non–human participants research by the University of California Irvine; the study used publicly available, deidentified data and therefore did not require informed consent. Reporting follows the Strengthening the Reporting of Observational Studies in Epidemiology (STROBE) guideline for cross-sectional studies.

### Data and Outcomes

Our primary data source was the 2016 to 2022 ACS.^13^ The ACS is a national- and state-representative ongoing household survey that collects information on demographic, economic, and social characteristics for approximately 1% of the US population annually. Since we were unable to directly observe undocumented status in the ACS, our analysis assumed that coverage changes among noncitizens would reflect changes among the subpopulation of undocumented individuals gaining eligibility under the expansion. Binary outcome variables included any, Medicaid, and private health insurance coverage. Coverage information was reported as of the interview date, and respondents could report multiple coverage sources. Respondents were instructed that they should not report single service plans or accident and disability insurance, but they were not explicitly told to exclude emergency coverage such as restricted-scope Medi-Cal. The ACS imputes all missing information.

We also examined monthly administrative data from the California Department of Health Care Services (DHCS) on the number of young adults aged 19 to 25 years who gained full-scope Medi-Cal coverage under the expansion through 2022.^14,15^ DHCS data are administratively measured in comparison with self-report coverage outcomes in the ACS, providing a reference point for our main ACS findings.

### Statistical Analysis

Our primary analysis used a triple difference regression model to compare trends in health insurance coverage among noncitizens aged 19 to 25 and 26 to 32 years before and after the California Medi-Cal expansion relative to 6 comparison states (Arizona, Florida, Illinois, Nevada, New York, and Texas) (eAppendix 1 and eAppendix 2 in Supplement 1). We selected comparison states that contained at least 1 county with an undocumented population of 100 000 or greater to align with the large undocumented population in California and to provide adequate samples of undocumented young adults. None of the comparison states had implemented a policy similar to California’s expansion by the end of 2022. Our analysis leveraged the age-based nature of California’s expansion because undocumented adults aged 26 years and older had not gained access to full-scope Medi-Cal by the end of our study period. This strategy accounted for differential trends by age that were similar across California and the comparison states by including both age- and state-based comparison groups for whom trends in coverage would be expected to evolve similarly absent the policy.

The independent variable of interest was the 3-way interaction between binary indicators for (1) California, (2) interview dates after Medi-Cal expansion, and (3) young adults aged 19 to 25 years. The coefficient on this interaction represented the triple difference estimate of the association of the expansion with outcomes among young adults aged 19 to 25 years compared with young adults aged 26 to 32 years in California relative to the comparison states. Our main analysis excluded data for 2020 given concerns about data quality issues,^16^ and therefore the postpolicy period included 2021 to 2022. Models controlled for single year of age dummies, public use microdata area (PUMA) fixed effects, and year fixed effects. PUMA fixed effects, which often align closely with county lines, accounted for time-invariant factors that varied by geography such as differences in local health care supply and resources for immigrant communities.^17^ Year fixed effects accounted for secular outcome trends. Given that our triple difference strategy leveraged variation across 3 dimensions (geography, age, and time), we also controlled for all 2-way interactions between the indicators for California, the postpolicy period, and the 19- to 25-year group. These interactions allowed for flexible differences between California and the comparison states before and after policy implementation and across the age groups, as well as differences by age before and after policy implementation.

Models also controlled for demographic characteristics including sex, race and ethnicity, and marital status. Race and ethnicity categories were based on respondent self-report and included Asian non-Hispanic, Black non-Hispanic, Hispanic, White non-Hispanic, and other non-Hispanic races. The latter included American Indian or Alaska Native, 2 major races, 3 or more major races, and any race or ethnicity not otherwise specified. We controlled for race and ethnicity to account for known disparities in health insurance coverage. Our main analysis did not control for educational attainment given that education is often incomplete before age 25 years. Regressions used ACS-provided survey weights, and errors were clustered at the PUMA level given important county-level programs and policies related to immigrant health. Regressions were estimated using ordinary least squares.

While Medi-Cal eligibility is income-based, our main analysis did not restrict the sample by income for 2 reasons. First, young adults aged 19 to 25 years are more likely to reside with their parents than adults aged 26 to 32 years, making it challenging to accurately assign income eligibility to some treatment group respondents (eg, family-level income measures in the ACS would include all coresiding family members). Second, there is evidence that expanding private parental coverage to adults aged 19 to 25 years under the Patient Protection and Affordable Care Act dependent coverage provision affected living arrangements,^18^ employment,^19^ and financial status.^20^ While we studied Medi-Cal expansion to a specific subpopulation of young adults, some of the same mechanisms may apply (eg, job lock).

We estimated regressions stratified by subgroup characteristics including race and ethnicity, sex, and age. For subgroup analyses by race and ethnicity, we grouped adults who identified as non-Hispanic Black and non-Hispanic other race due to smaller sample sizes. When examining results by age, we included only treatment group observations in the target age range but all control group observations (ie, ages 26-32 years).

To examine the validity of the parallel trends assumption, we estimated event study models that interacted each prepolicy year with the indicators for California vs the comparison states and for those aged 19 to 25 years vs 26 to 32 years. All other model controls remained the same. We examined several other sensitivity analyses: (1) including and excluding data from 2020 and 2021, (2) using a health insurance hierarchy, (3) including completed education controls, (4) restricting analyses to respondents residing without parents who had household incomes up to 138% of the federal poverty level, restricting analyses to PUMAs in the top quartile in terms of the number of noncitizen young adults with incomes up to 138% of the federal poverty level, and estimating difference-in-differences models that compared California noncitizens who were aged 19 to 25 and 26 to 32 years, before and after policy implementation.

We conducted a post hoc analysis to better understand the scale of our estimates on the newly eligible population since our analysis included all noncitizens aged 19 to 25 years and not only those who gained full-scope Medi-Cal eligibility. This calculation assumed that 42% of noncitizens were undocumented based on Pew Research Center estimates of the undocumented and noncitizen population sizes,^21^ and that 41% of noncitizens aged 19 to 25 years had incomes up to 138% of the federal poverty level based on our estimates from the ACS. Dividing our ACS estimates by the product of these 2 percentages provided a rough estimate for undocumented, income-eligible young adults. To translate these results into the number of young adults gaining coverage, we used Migration Policy Institute population estimates for California in 2019.^22^

Estimates were considered statistically significant for a 2-sided *P* value less than .05. Estimates mentioned in the text are statistically significant unless otherwise noted. The analysis was performed using Stata SE version 17 (Stata Corp) from January 2024 to August 2025.^23^

## Results

### Sample Descriptive Statistics and Outcome Trends

The final sample included 19 773 noncitizen ACS respondents aged 19 to 25 years and 32 515 noncitizen ACS respondents aged 26 to 32 years, residing in California, and 28 535 adults aged 19 to 25 years and 43 213 adults aged 26 to 32 years residing in comparison states. These unweighted samples translated to weighted California sample sizes of 2 341 702 aged 19 to 25 years and 3 993 584 aged 26 to 32 years and weighted comparison state sample sizes of 3 851 595 aged 19 to 25 years and 6 167 401 aged 26 to 32 years. Baseline weighted percentages for the 19- to 25-year treatment group included 52.1% (95% CI, 51.0%-53.2%) male, 31.9% (95% CI, 30.7%-33.0%) Asian non-Hispanic, 1.8% (95% CI, 1.5%-2.2%) Black non-Hispanic, 54.6% (95% CI, 53.4%-55.9%) Hispanic, 9.7% (95% CI, 8.9%-10.5%) White non-Hispanic, and 2.0% (95% CI, 1.6%-2.3%) other race non-Hispanic (Table 1). Baseline demographic characteristics were similar among those aged 19 to 25 years and 26 to 32 years, with the exception of married status (eg, among California residents: 15.9%; 95% CI, 15.1%-16.7% for those aged 19 to 25 years vs 51.5%; 95% CI, 50.6%-52.4% for those aged 26 to 32 years).

**Table 1.** Summary Statistics by Age and State of Residence, American Community Survey, 2016-2022

Statistic	Share of sample, mean (SE) [estimated subpopulation size]^a^							
	California				Comparison states			
	Age 19-25 y		Age 26-32 y		Age 19-25 y		Age 26-32 y	
	2016-2019	2021-2022	2016-2019	2021-2022	2016-2019	2021-2022	2016-2019	2021-2022
Demographics								
Sex								
Male	0.521 (0.006) [857 887]	0.523 (0.008) [363 760]	0.513 (0.004) [1 456 542]	0.522 (0.006) [603 511]	0.521 (0.005) [1 396 660]	0.529 (0.007) [620 111]	0.518 (0.003) [2 246 555]	0.522 (0.005) [957 481]
Female	0.479 (0.006) [788 762]	0.477 (0.008) [331 293]	0.487 (0.004) [1 381 333]	0.478 (0.006) [552 198]	0.479 (0.005) [1 282 057]	0.471 (0.007) [552 767]	0.482 (0.003) [2 087 917]	0.478 (0.005) [875 448]
Race and ethnicity								
Asian non-Hispanic	0.319 (0.006) [524 599]	0.297 (0.008) [206 607]	0.305 (0.004) [866 300]	0.345 (0.007) [398 328]	0.211 (0.004) [564 781]	0.181 (0.005) [212 006]	0.215 (0.003) [930 861]	0.212 (0.005) [389 337]
Black non-Hispanic	0.018 (0.002) [29 998]	0.018 (0.002) [12 470]	0.016 (0.001) [46 584]	0.016 (0.002) [17 999]	0.096 (0.003) [256 537]	0.096 (0.005) [112 023]	0.077 (0.002) [333 815]	0.077 (0.004) [141 147]
Hispanic	0.546 (0.006) [899 692]	0.589 (0.009) [409 671]	0.559 (0.004) [1 585 565]	0.519 (0.007) [599 646]	0.570 (0.005) [1 528 039]	0.604 (0.008) [707 924]	0.566 (0.004) [2 454 712]	0.574 (0.006) [1 052 523]
White non-Hispanic	0.097 (0.004) [159 571]	0.075 (0.005) [52 015]	0.102 (0.003) [288 481]	0.097 (0.004) [112 284]	0.102 (0.003) [272 217]	0.089 (0.005) [104 912]	0.121 (0.003) [524 117]	0.107 (0.003) [196 885]
Other race^b^	0.020 (0.002) [32 789]	0.021 (0.003) [14 290]	0.018 (0.001) [50 945]	0.024 (0.002) [27 452]	0.021 (0.001) [57 143]	0.031 (0.003) [36 013]	0.021 (0.001) [90 967]	0.029 (0.002) [53 037]
Married	0.159 (0.004) [261 932]	0.134 (0.006) [93 460]	0.515 (0.004) [1 460 855]	0.480 (0.007) [554 251]	0.192 (0.004) [513 966]	0.175 (0.006) [204 824]	0.513 (0.004) [2 224 435]	0.494 (0.006) [906 182]
Dependent variables								
Any health insurance coverage	0.737 (0.005) [1 213 634]	0.750 (0.008) [521 496]	0.738 (0.004) [2 094 478]	0.767 (0.006) [886 484]	0.575 (0.005) [1 540 272]	0.564 (0.008) [660 958]	0.575 (0.004) [2 492 270]	0.597 (0.006) [1 093 823]
Medicaid	0.270 (0.005) [444 073]	0.307 (0.008) [213 306]	0.221 (0.004) [627 324]	0.199 (0.005) [230 311]	0.136 (0.003) [364 270]	0.146 (0.005) [171 547]	0.115 (0.002) [500 207]	0.119 (0.004) [217 292]
Private health insurance	0.475 (0.006) [782 168]	0.463 (0.009) [321 622]	0.524 (0.004) [1 485 774]	0.577 (0.007) [666 358]	0.449 (0.005) [1 202 321]	0.428 (0.007) [502 122]	0.466 (0.004) [2 021 705]	0.486 (0.006) [891 083]
Estimated population, No.	1 646 649	695 053	2 837 875	1 155 709	2 678 717	1 172 878	4 334 472	1 832 929
Observations, No.	13 549	6224	22 345	10 170	19 577	8958	29 779	13 434

^a^ Population means were estimated using American Community Survey sampling weights. Standard errors in parenthesis correct for complex survey design. Year 2020 was omitted due to data quality concerns following the COVID-19 pandemic.

^b^ Other includes respondents who identified as American Indian or Alaska Native, 2 major races, 3 or more major races, or any race or ethnicity not otherwise specified.

Among California residents aged 19 to 25 years at baseline, 73.7% (95% CI, 72.6%-74.8%) reported any health insurance, 27.0% (95% CI, 25.9% 28.0%) reported Medicaid coverage, and 47.5% (95% CI, 46.3%-48.7%) reported private coverage. As noted previously, we expected that baseline reported Medicaid coverage might include restricted-scope Medi-Cal.

The Figure presents unadjusted trends in Medicaid coverage by age and California residence from the ACS (Figure, A) and administrative data on Medi-Cal enrollment under the new policy from DHCS (Figure, B). ACS trends in Medicaid enrollment appeared similar and relatively flat for the treatment and control groups from 2016 to 2019 (Figure, A). Medicaid enrollment increased among California residents aged 19 to 25 years from 2020 to 2022, with progressive increases across the 3 post–policy implementation years. Changes in Medicaid enrollment among the unaffected groups were less pronounced.

**Figure.**
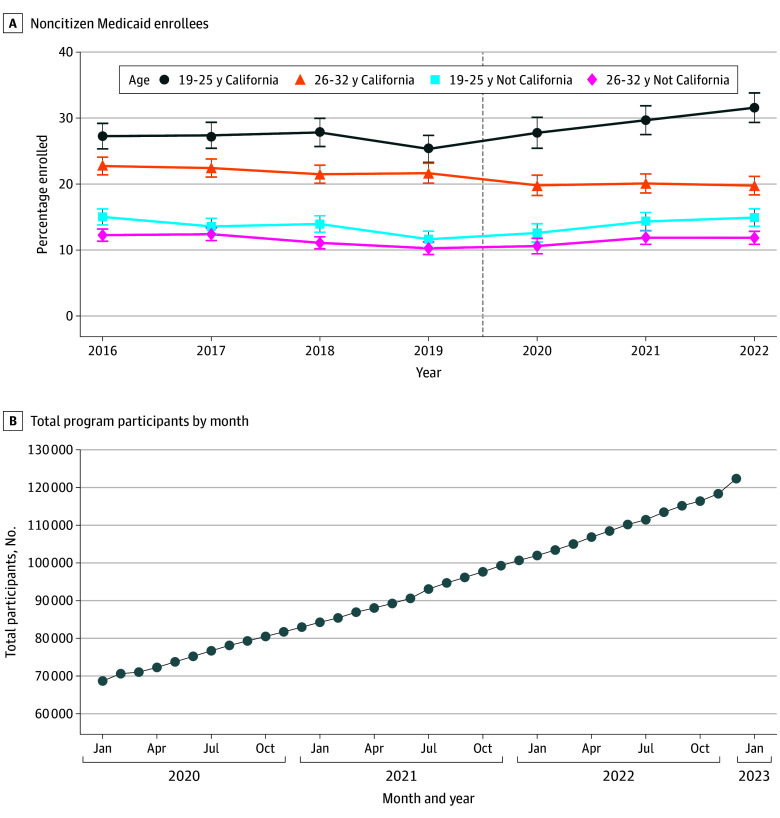
Line Graphs of Medicaid Enrollment Among Young Adult Noncitizens, American Community Survey and California Department of Health Care Services A, Weighted Medicaid participation rates in the 2016-2022 American Community Survey for treatment (19-25 years) and control groups (26-32 years) for California and non-California states with large undocumented populations. The vertical dashed line indicates the policy’s implementation. B, Total full-scope Medi-Cal participation counts for the state of California from the California Department of Health Care Services for January 2020 to December 2022. These counts include adults aged 19 to 25 years who newly enrolled in coverage under California’s expansion. The counts include undocumented young adults and a small number of nonpregnant documented noncitizens aged 21 to 25 years who were not eligible for federal funding before 2020 (eg, student visa holders).

According to DHCS data, initial enrollment under the expansion (68 690 enrollees) was similar to prepolicy restricted-scope Medi-Cal enrollment in September 2019 (67 792 enrollees),^14^ indicating that former restricted-scope enrollees likely accounted for most new enrollment during the initial months following expansion (Figure, B).^15^ Full-scope Medi-Cal enrollment continued to grow linearly throughout 2020 to 2022, reaching 122 365 young adults by the end of 2022.

### Regression Results

Table 2 presents our main triple difference results. We estimated that Medi-Cal expansion was associated with a 4.2 (95% CI, 1.3-7.1)–percentage point increase in Medicaid coverage. This finding was in line with unadjusted analyses in Table 1 and the Figure. We also estimated that Medi-Cal expansion was associated with a 3.5 (95% CI, 0.2-6.8)–percentage point increase in any coverage. Our estimate for private insurance was small in magnitude and not statistically significant.

**Table 2.** Triple Difference Estimates of Medi-Cal Expansion and Health Insurance Outcomes, American Community Survey, 2016-2022

Estimate	Percentage point change in outcome per 100 (SE)^a^		
	Any coverage	Medicaid	Private health insurance
California residents aged 19-25 y post–policy implementation	0.035 (0.017)^b^	0.042 (0.015)^c^	0.002 (0.017)
Male sex	−0.069 (0.003)^d^	−0.072 (0.003)^d^	0.001 (0.003)
Race and ethnicity			
Asian non-Hispanic	0.067 (0.006)^d^	−0.029 (0.005)^d^	0.097 (0.007)^d^
Black non-Hispanic	−0.041 (0.011)^d^	0.066 (0.008)^d^	−0.104 (0.011)^d^
Hispanic	−0.257 (0.006)^d^	0.064 (0.005)^d^	−0.321 (0.007)^d^
Other race^e^	−0.021 (0.013)	0.054 (0.012)^d^	−0.077 (0.015)^d^
Married	0.043 (0.004)^d^	0.020 (0.003)^d^	0.022 (0.004)^d^
Observations, No.	124 036	124 036	124 036

^a^ All regressions are run on samples from 2016 to 2022, excluding 2020, due to data concerns arising from the COVID-19 pandemic.

^b^ *P* < .05.

^c^ *P* < .01.

^d^ *P* < .001.

^e^ Other includes respondents who identified as American Indian or Alaska Native, 2 major races, 3 or more major races, or any race or ethnicity not otherwise specifed.

In subgroup analyses by race and ethnicity, only Hispanic young adults experienced a significant increase in Medicaid coverage (6.7 [95% CI, 2.6-10.9] percentage points) (Table 3). Estimated percentage-point increases in Medicaid coverage by sex (males: 3.6 [95% CI, 0.1-7.1] percentage points; females: 5.0 [95% CI, 0.7-9.3] percentage points) and by age (19 to 22 years: 4.4 [95% CI, 0.7-8.1] percentage points; 23 to 25 years: 4.0 [95% CI, 0.7-7.3] percentage points) were not statistically different from one another. Triple difference estimates for any coverage were positive for most groups but generally not statistically significant at conventional levels, with the exception of young adults aged 19 to 22 years (4.4 [95% CI, 0.5-8.3] percentage points).

**Table 3.** Triple Difference Estimates by Demographic Characteristics, American Community Survey, 2016-2022

Estimate by demographic	Percentage point change in outcome per 100 (SE)^a^		
	Any coverage	Medicaid	Private health insurance
**Race and ethnicity**			
Asian non-Hispanic			
California residents aged 19-25 y post–policy implementation	−0.013 (0.023)	0.000 (0.022)	−0.007 (0.029)
Observations, No.	37 057	37 057	37 057
Black and other race, non-Hispanic^b^			
California residents aged 19-25 y post–policy implementation	0.006 (0.076)	0.007 (0.075)	0.006 (0.090)
Observations, No.	8708	8708	8708
Hispanic			
California residents aged 19-25 y post–policy implementation	0.047 (0.025)^c^	0.067 (0.021)^d^	−0.007 (0.024)
Observations, No.	63 880	63 880	63 880
White non-Hispanic			
California residents aged 19-25 y post–policy implementation	0.037 (0.052)	−0.025 (0.047)	0.081 (0.061)
Observations, No.	14 391	14 391	14 391
Sex			
Male			
California residents aged 19-25 y post–policy implementation	0.035 (0.024)	0.036 (0.018)^e^	0.005 (0.024)
Observations, No.	63 106	63 106	63 106
Female			
California residents aged 19-25 y post–policy implementation	0.033 (0.023)	0.050 (0.022)^e^	−0.003 (0.024)
Observations, No.	60 930	60 930	60 930
Age, y^f^			
19-22			
California residents post–policy implementation	0.044 (0.020)^e^	0.044 (0.019)^e^	0.012 (0.021)
Observations	100 825	100 825	100 825
23-25			
California residents post–policy implementation	0.027 (0.020)	0.040 (0.017)^e^	−0.007 (0.021)
Observations, No.	98 939	98 939	98 939

^a^ All regressions are run on samples from 2016 to 2022, excluding 2020, due to data concerns arising from the COVID-19 pandemic.

^b^ Included respondents who identified as American Indian or Alaska Native, 2 major races, 3 or more major races, or any race not otherwise specified.

^c^ *P* = .06.

^d^ *P* < .01.

^e^ *P* < .05.

^f^ Restricted the treated group to individuals in the specified age range, keeping the full sample of noncitizens aged 26 to 32 years as a control group.

In post hoc analyses, our results translated to a 24.4–percentage-point gain in Medi-Cal coverage (ie, 4.2/[0.41 × 0.42]) and a 20.3–percentage-point increase in any coverage (ie, 3.5/[0.41 × 0.42]) among newly eligible young adults. Based on population estimates from the Migration Policy Institute,^22^ this translated to 30 665 more young adults enrolled in Medi-Cal and 25 554 more young adults with any coverage.

### Sensitivity Analyses

Our event study analysis generally did not suggest significant differential preexisting outcome trends (eFigures 1-3 in Supplement 1). Results were similar when we included data for 2020 or alternatively excluded data for 2021 (eTable 1 in Supplement 1), when we used health insurance hierarchies (eTable 2 in Supplement 1), and when we included controls for completed education (eTable 3 in Supplement 1). Results were also qualitatively similar when we excluded all demographic controls (eTable 4 in Supplement 1) and when we restricted the sample by income or by residence in a low-income PUMA (eTable 5 in Supplement 1), although some estimates were less precise. Finally, difference-in-differences analyses restricted to California noncitizen adults suggested a significant increase in Medi-Cal coverage, a decrease in private insurance, and a small and statistically nonsignificant change in the uninsured rate (eTable 6 in Supplement 1). This alternative strategy did not account for differential trends by age group among noncitizens that are similar across California and the comparison states. As shown in Table 1, there was an increase in coverage among those aged 26 to 32 years before and after 2020 across California and the control states, which is accounted for in our triple difference estimates.

## Discussion

Eight states have opted to provide state-funded coverage to some groups of adults regardless of immigration status, with many of these expansions occurring between 2020 and 2025.^4^ Several states, including California, plan to pause or end these programs due to funding constraints.^4^ For example, California’s Medi-Cal program experienced a $6.2 billion budget shortfall in fiscal year 2024-2025, driven in part by unexpectedly high enrollment under expansions to undocumented adults in 2020, 2022, and 2024.^24^ Such budget shortfalls could be exacerbated by new policies intended to reduce federal Medicaid spending.^25,26^ The impacts of these decisions are uncertain given limited evidence evaluating recent policies to insure undocumented adults.

This cross-sectional study found that the California 2020 Medi-Cal expansion to young adults aged 19 to 25 years regardless of immigration status was associated with significant increases in any and Medi-Cal coverage. Relative to baseline rates among California residents aged 19 to 25 years, our estimates represent a 15.5% increase in Medicaid coverage and 4.7% increase in any coverage. In post hoc analyses, these gains translated to 30 665 more young adults enrolled in Medi-Cal and 25 554 more young adults with any coverage due to the expansion. Our main analysis did not suggest a significant change in private coverage, indicating that many young adults who gained Medi-Cal were uninsured prior to the expansion. The qualitative pattern of these results was consistent with an analysis of the California 2016 expansion to undocumented children,^9^ and indicated that the newly eligible may have had fewer private insurance options.

Our estimated increase in Medi-Cal enrollment was below that from DHCS (about 100 686 enrollees at the end of 2021, the midpoint of our post–policy analysis period). To the extent that respondents with restricted-scope coverage reported that they were uninsured in the prepolicy period, transitions from restricted- to full-scope Medi-Cal may have contributed to our estimated increase in any coverage. However, because ACS respondents were not explicitly instructed to exclude restricted-scope Medi-Cal as a coverage source, our analysis likely failed to capture many of these transitions and may more accurately represent changes in any Medi-Cal coverage (restricted- or full-scope). It is also plausible that the estimate of California’s undocumented population used to translate our triple difference estimates into population sizes underestimated the true number, which could also explain part of the gap. Because restricted-scope enrollees only had coverage for emergency services prior to the expansion, gaining full-scope Medi-Cal eligibility has substantial potential implications for coverage and access.

Subgroup analyses suggested that Hispanic young adults experienced the largest increase in Medi-Cal coverage, although confidence intervals for non-Hispanic White and adults who reported other race were wide due to smaller sample sizes. Hispanic adults are among the most likely race and ethnicity group in the US to be uninsured and constitute the majority of the undocumented population.^3,27^ These findings indicate that the expansion may have helped to close coverage gaps by ethnicity. Implications for disparities in health and well-being by race and ethnicity warrant further attention.

### Limitations

This study had several limitations. First, health insurance coverage was self-reported in the ACS and Medicaid coverage is generally undercounted.^28^ Moreover, noncitizen individuals may be less likely to respond to surveys such as the ACS. Second, we used noncitizen status to identify young adults likely to gain eligibility under California’s expansion given data limitations. Our analysis assumed that noncitizen status would serve as a reasonable proxy for undocumented status in examining coverage changes before and after policy implementation. We conducted post hoc analyses to translate our estimates to newly eligible young adults, although all estimates were approximate. Third, we could not distinguish restricted- and full-scope Medi-Cal, and our analysis likely did not accurately capture these transitions. Fourth, similar to all observational studies, our analysis could not account for all policy and population factors potentially co-occurring with California’s expansion. For example, all states suspended the use of Medicaid eligibility redeterminations from 2020 to 2023 under the Families First Coronavirus Response Act. Given our study design, such confounders would have to differentially affect California noncitizens aged 19 to 25 years and 26 to 32 years relative to those same groups in other states to bias our estimates. Fifth, because California’s immigrant health infrastructure and safety-net programs differ substantially from other states, and because this policy targeted an age range experiencing life-cycle transitions (eg, school completion and labor market entry), the degree to which our estimates translate to other states or wider policy expansions may be limited.

## Conclusions

In this cross-sectional study of the California 2020 Medi-Cal expansion to young adults aged 19 to 25 years regardless of immigration status, we found that expansion was associated with statistically significant increases in Medicaid and any coverage without corresponding reductions in private coverage for California noncitizens aged 19 to 25 years. In addition, DHCS enrollment data are suggestive of a large shift from restricted-scope, emergency-only coverage to full-scope coverage,^15^ which is likely to improve health care access and preventive care use. Moreover, uninsurance is associated with higher rates of avoidable emergency department use.^29^ Expanding access to preventive care may reduce reliance on the emergency department and could also have downstream health benefits.^30^ Further research is needed to understand the full scope of the benefits and costs of expansion and how they evolve over time as newly insured adults gain more experience in interacting with the health care system.

## Notes

Supplement 1. eAppendix 1. Regression Specification eAppendix 2. Descriptions of Supplementary Data eFigure 1. Event Study Plot of the Effects of California’s Expansion to Undocumented 19-25-Year-Olds on Whether an Individual Reports Having Any Health Insurance Coverage eFigure 2. Event Study Plot of the Effects of California’s Expansion to Undocumented 19-25-Year-Olds on Whether an Individual Reports Having Health Insurance Coverage Through Medicaid eFigure 3. Event Study Plot of the Effects of California’s Expansion to Undocumented 19-25-Year-Olds on Whether an Individual Reports Having Private Health Insurance Coverage eTable 1. Triple Difference Estimates of the Effects of California’s Expansion to Undocumented 19-25-Year-Olds on Main Health Insurance Outcomes, Sensitivity to Inclusion/Exclusion of Potentially Contaminated Data Years eTable 2. Triple Difference Estimates of the Effects of California’s Expansion to Undocumented 19-25-Year-Olds on Health Insurance Outcomes Defined by SHADAC and KFF Hierarchies eTable 3. Triple Difference Estimates of Medi-Cal Expansion on Health Insurance Outcomes, Sensitivity Analysis of Inclusion of Educational Attainment eTable 4. Triple Difference Estimates of Medi-Cal Expansion on Health Insurance Outcomes, Sensitivity Analysis of Exclusion of Demographic Controls eTable 5. Triple Difference Regression Estimates of the Effects of California’s Expansion to Undocumented 19-25-Year-Olds on Main Health Insurance Outcomes, Sensitivity to Restricting Sample to Disadvantaged Economic Subgroups eTable 6. Difference-in-Differences Estimates of Medi-Cal Expansion on Health Insurance Outcomes, California Only, ACS 2016-2022

Supplement 2. Data Sharing Statement
