# Supplementary material for: The California 2020 Medi-Cal Expansion to Young Adults and Coverage Among Noncitizens
Source: JAMA Netw Open. 2026 May 13;9(5):e2612332. doi: 10.1001/jamanetworkopen.2026.12332 (PMC13173380; doi:10.1001/jamanetworkopen.2026.12332)
Supplement: Supplement 1. — eAppendix 1. Regression Specification eAppendix 2. Descriptions of Supplementary Data eFigure 1. Event Study Plot of the Effects of California’s Expansion to Undocumented 19-25-Year-Olds on Whether an Individual Reports Having Any Health Insurance Coverage eFigure 2. Event Study Plot of the Effects of California’s Expansion to Undocumented 19-25-Year-Olds on Whether an Individual Reports Having Health Insurance Coverage Through Medicaid eFigure 3. Event Study Plot of the Effects of California’s Expansion to Undocumented 19-25-Year-Olds on Whether an Individual Reports Having Private Health Insurance Coverage eTable 1. Triple Difference Estimates of the Effects of California’s Expansion to Undocumented 19-25-Year-Olds on Main Health Insurance Outcomes, Sensitivity to Inclusion/Exclusion of Potentially Contaminated Data Years eTable 2. Triple Difference Estimates of the Effects of California’s Expansion to Undocumented 19-25-Year-Olds on Health Insurance Outcomes Defined by SHADAC and KFF Hierarchies eTable 3. Triple Difference Estimates of Medi-Cal Expansion on Health Insurance Outcomes, Sensitivity Analysis of Inclusion of Educational Attainment eTable 4. Triple Difference Estimates of Medi-Cal Expansion on Health Insurance Outcomes, Sensitivity Analysis of Exclusion of Demographic Controls eTable 5. Triple Difference Regression Estimates of the Effects of California’s Expansion to Undocumented 19-25-Year-Olds on Main Health Insurance Outcomes, Sensitivity to Restricting Sample to Disadvantaged Economic Subgroups eTable 6. Difference-in-Differences Estimates of Medi-Cal Expansion on Health Insurance Outcomes, California Only, ACS 2016-2022 [file jamanetwopen-e2612332-s001.pdf]

# Supplemental Online Content

Leonard R, Lipton BJ. The California 2020 Medi-Cal expansion to young adults and coverage among noncitizens. *JAMA Netw. Open*. 2026;9(5):e2612332. doi:10.1001/jamanetworkopen.2026.12332

**eAppendix 1.** Regression Specification

**eAppendix 2.** Descriptions of Supplementary Data

**eFigure 1.** Event Study Plot of the Effects of California's Expansion to Undocumented 19-25-Year-Olds on Whether an Individual Reports Having Any Health Insurance Coverage

**eFigure 2.** Event Study Plot of the Effects of California's Expansion to Undocumented 19-25-Year-Olds on Whether an Individual Reports Having Health Insurance Coverage Through Medicaid

**eFigure 3.** Event Study Plot of the Effects of California's Expansion to Undocumented 19-25-Year-Olds on Whether an Individual Reports Having Private Health Insurance Coverage

**eTable 1.** Triple Difference Estimates of the Effects of California's Expansion to Undocumented 19-25-Year-Olds on Main Health Insurance Outcomes, Sensitivity to Inclusion/Exclusion of Potentially Contaminated Data Years

**eTable 2.** Triple Difference Estimates of the Effects of California's Expansion to Undocumented 19-25-Year-Olds on Health Insurance Outcomes Defined by SHADAC and KFF Hierarchies

**eTable 3.** Triple Difference Estimates of Medi-Cal Expansion on Health Insurance Outcomes, Sensitivity Anlaysis of Inclusion of Educational Attainment

**eTable 4.** Triple Difference Estiamtes of Medi-Cal Expansion on Health Insurance Outcomes, Sensitivity Analysis of Exclusion of Demographic Controls

**eTable 5.** Triple Difference Regression Estimates of the Effects of California's Expansion to Undocumented 19-25-Year-Olds on Main Health Insurance Outcomes, Sensitivity to Restricting Sample to Disadvantaged Economic Subgroups

**eTable 6.** Difference-in-Differences Estimates of Medi-Cal Expansion on Health Insurance Outcomes, California Only, ACS 2016-2022

This supplemental material has been provided by the authors to give readers additional information about their work.

© 2026 Leonard R et al. *JAMA Network Open*.

**eAppendix 1. Regression Specification**

As described in the main text, our primary specification is a triple difference approach, comparing trends in health insurance coverage among noncitizens ages 19-25 and 26-32 before and after California's Medi-Cal expansion in California relative to 6 comparison states (Arizona, Florida, Illinois, Nevada, New York, and Texas). This empirical model can be expressed as follows:

$$Y_{itc} = \alpha_{itc} + \beta_1 1925_i \cdot Post_t \cdot CA_c + \beta_2 Post_t \cdot 1925_i + \beta_3 Post_t \cdot CA_c + \beta_4 CA_c \cdot 1925_i + \boldsymbol{\beta_5 X_i} + \varphi_t + \eta_c + \varepsilon_{itc} \quad (1)$$

$1925_i$ equals one if the individual is a 19-25-year-old and zero otherwise. $Post_t$ equals one for observations in years 2021-2022 and zero otherwise. $CA_c$ equals one for public-use microdata areas (PUMAs) in California and zero otherwise. The interaction of these three variables makes $\beta_1$ the triple difference coefficient of interest when the sample is restricted to only 19-32-year-old noncitizens in California and the chosen comparison states. $X_i$ is a vector containing the individual-level demographic controls featured in Panel I of Table 1, plus indicator variables for age. The inclusion of year fixed effect $\varphi_t$ and PUMA fixed effect $\eta_c$ provide the interpretation of $\beta_1$ as "within" PUMA and year. PUMA fixed effects account for time-invariant characteristics that may be associated with coverage outcomes among noncitizen adults, including long-standing indigent care programs in California that provided coverage options regardless of citizenship status to some adults. All regressions are weighted using the ACS-provided survey weights, making the resulting estimates at the population level. Standard errors are clustered at the PUMA level.

© 2026 Leonard R et al. *JAMA Network Open*.

## eAppendix 2. Description of Tables and Figures

**e**Table 1 tests the sensitivity of our primary specification to the inclusion/exclusion of data years 2020 and 2021, which may have been affected by the COVID-19 pandemic. Panel I contains the results of fitting equation (1) on California and comparison state ACS data for noncitizens for data years 2016-2022 (i.e., it adds 2020 data to the sample in Table 2). This inclusion does not significantly affect the results for any of the three primary outcomes. Panel II fits equation (1) on data years 2016-2020 and 2022 (i.e., it excludes 2021 instead of 2020). Again, this produces results that are functionally equivalent to the results in Table 2. Panel III fits equation (1) on data years 2016-2019 and 2022 (i.e., it excludes *both* 2020 and 2021 from the analysis. This also produces estimates that are consistent with the main results in Table 2.

eTable 2 presents triple difference regression estimates of the effects of California's expansion to undocumented 19-25-year-olds on health insurance outcomes defined by the State Health Access Data Assistance Center (SHADAC) and Kaiser Family Foundation (KFF) hierarchies. These hierarchies account for the fact that it is common for individuals to have multiple sources of coverage by identifying the likely "primary" source of coverage when individuals report more than one source. These hierarchy-driven variables are mutually exclusive indicator variables that equal one for the attributed "primary" coverage source, and zero for all other sources. The SHADAC outcomes are coded as: Medicare equals one if the individual reports health insurance through Medicare; Employer/Military equals one if the individual reports coverage through their employer or through the military (VA or TRICARE) and does not report coverage through Medicare; Medicaid equals one if the individual reports coverage through Medicaid and does not report coverage through

© 2026 Leonard R et al. *JAMA Network Open*.

Medicare, their employer, or the military; Direct Purchase equals one if the individual reports coverage through a private health insurance plan purchased directly by them or a family member and does not report coverage through Medicare, the military, their employer, or Medicaid; No Coverage equals one if the individual reports not having any health insurance coverage. Medicaid coverage included "Medicaid, Medical Assistance, or any other kind of government-assistance plan for those with low incomes or a disability." The KFF outcomes are coded as: Medicaid equals one if the individual reports coverage through Medicaid; Employer equals one if the individual reports coverage through their employer and does not report coverage through Medicaid; Medicare equals one if the individual reports health insurance through Medicare and does not report coverage through Medicaid or their employer; Military equals one if the individual reports health insurance through the military and does not report coverage through Medicaid, their employer, or Medicare; Direct Purchase equals one if the individual reports coverage through a private health insurance plan purchased directly by them or a family member and does not report coverage through Medicaid, their employer, Medicare, or the military; No Coverage equals one if the individual reports not having any health insurance coverage. Consistent with the primary estimates presented in Table 2, triple difference estimates based on the SHADAC and KFF hierarchies suggest that the expansion of Medi-Cal to cover young undocumented adults led to a reduction in uninsurance rates and a commensurate increase in Medi-Cal coverage rates.

eTable 3 test the sensitivity of the main triple difference estimates to inclusion of additional controls for educational attainment. The estimating equation for Table A3 is

© 2026 Leonard R et al. *JAMA Network Open*.

identical to that of Table 2 (and Table A1), except for the addition of the following regressors: Less than grade 12 equals one if the respondent identified as having any level of education below completion of grade twelve, and zero otherwise; Completed grade 12 equals one if the respondent identified as having completed grade twelve, and zero otherwise; 1-2 years college equals one if the respondent identified as having completed one to two years of college, and zero otherwise (the ACS variable “educ” from IPUMS does not include an option for having attended three years of college for our data years); 4 years college equals one if the respondent identified as having attended four years of college (obtained a bachelors degree), and zero otherwise; 5+ years college equals one if the respondent identified as having completed at least five years of college (obtained some professional or graduate degree), and zero otherwise. Results in Table A3 suggest that the main estimates in Table 2 are not sensitive to the inclusion or exclusion of controls for educational attainment.

eTable 4 tests the sensitivity of the main triple difference estimates to the exclusion of all demographic controls. This regression specification estimates equation (1) above with only a set of age-specific indicators populating $\boldsymbol{X_i}$. Results in Table A4 suggest that the estimate of the expansion’s effect on undocumented 19-25-year-olds’ Medicaid enrollment in Table 2 is not sensitive to demographic controls, while the estimate on any coverage is sensitive to demographic controls.

eTable 5 presents triple difference regression estimates of the effects of California’s expansion to undocumented 19-25-year-olds on our main health insurance outcomes for disadvantaged economic subgroups. These estimates examine the extent to which this

© 2026 Leonard R et al. *JAMA Network Open*.

policy affected low-income individuals who do not live with at least one parent (Panel I), individuals living in relatively low-income noncitizen counties (Panel II), and low-income individuals living in relatively high-income noncitizen counties (Panel III). We define low-income individuals as having total family income less than or equal to 138% of the federal poverty threshold based on the IPUMS USA variable "Poverty", which calculated family income as a percentage of the family's poverty threshold. We define relatively low-income noncitizen counties as counties in the top quartile for the percentage of 19-32-year-old noncitizens who are low-income. To construct this relatively low-income noncitizen county variable, we calculate the simple, county-level mean of an indicator for poverty status among 19-32-year-old noncitizens and create an individual-level indicator equal to one if the individual lives in a county for which calculated 19-32-year-old noncitizen poverty rate falls in the top quartile; for relatively high-income counties, we select the bottom quartile.

eTable 6 presents difference-in-differences (DD) estimates of the effects of California's expansion to undocumented 19-25-year-olds on our main health insurance outcomes, comparing trends in insurance uptake among noncitizen 19-25-year-olds in California to those of 26-32-year-old noncitizens in California before and after Medi-Cal was expanded to 19-25-year-old noncitizens in 2020. This empirical model can be expressed as follows:

$$Y_{itc} = \alpha_{itc} + \gamma_1 1925_i \cdot Post_t + \gamma_2 1925_i + \gamma_4 \boldsymbol{X_i} + \varphi_t + \eta_c + \varepsilon_{itc} \quad (2)$$

$1925_i$ equals one if the individual is a 19-25-year-old and zero otherwise. $Post_t$ equals one for observations in years 2020-2022 and zero otherwise. Their interaction makes $\gamma_1$ the coefficient of interest when the sample is restricted to only 19-32-year-old noncitizens in

© 2026 Leonard R et al. *JAMA Network Open*.

California, identifying the differential effect of being a 19-25-year-old vs. 26-32-year-old noncitizen in California before and after the 2020 Medi-Cal expansion. Other relevant terms are the same as in equation (1). Similar to our main triple difference analysis, we estimate a significant increase in Medicaid coverage (5.4 percentage point increase). However, unlike our main analysis, the DD estimates presented in Table A6 suggest that the post-policy increase in Medi-Cal participation may have been driven by a reduction in private coverage—i.e., these estimates suggest that Medi-Cal crowds out private coverage. Since our main triple difference specification accounts for differential trends by age group that are similar across California and the control states, we prefer the triple difference estimates presented in the main text. As shown in main text Table 1, any coverage increases among 26-32 year-olds in both California and the control states before and after the policy's implementation. By excluding the comparison states as in Table A6, we do not account for this increase whereas it is netted out in our triple difference model, which explains the dampened estimate for any coverage in the difference-in-differences relative to triple difference analysis. Given that increasing any coverage among 26-32 year-olds occurs in comparison states as well as in California, it is likely unrelated to the California's Medi-Cal expansion.

eFigures 1, 2, and 3 present event study plots displaying the pre- and post-treatment dynamics of Any Coverage, Medicaid coverage, and Private HI coverage, respectively. These event studies represent visual falsification tests for the parallel trends assumption, which is the identifying assumption of a difference-in-differences (and triple difference) research design. None of these event study plots presents evidence that prior to treatment,

© 2026 Leonard R et al. *JAMA Network Open*.

conditional on observable characteristics, the health insurance trends of our treatment and control groups were not unfolding parallelly.

© 2026 Leonard R et al. *JAMA Network Open*.

eFigure 1: Event Study Plot of the Effects of California's Expansion to Undocumented 19-25-Year-Olds on Whether an Individual Reports Having Any Health Insurance Coverage

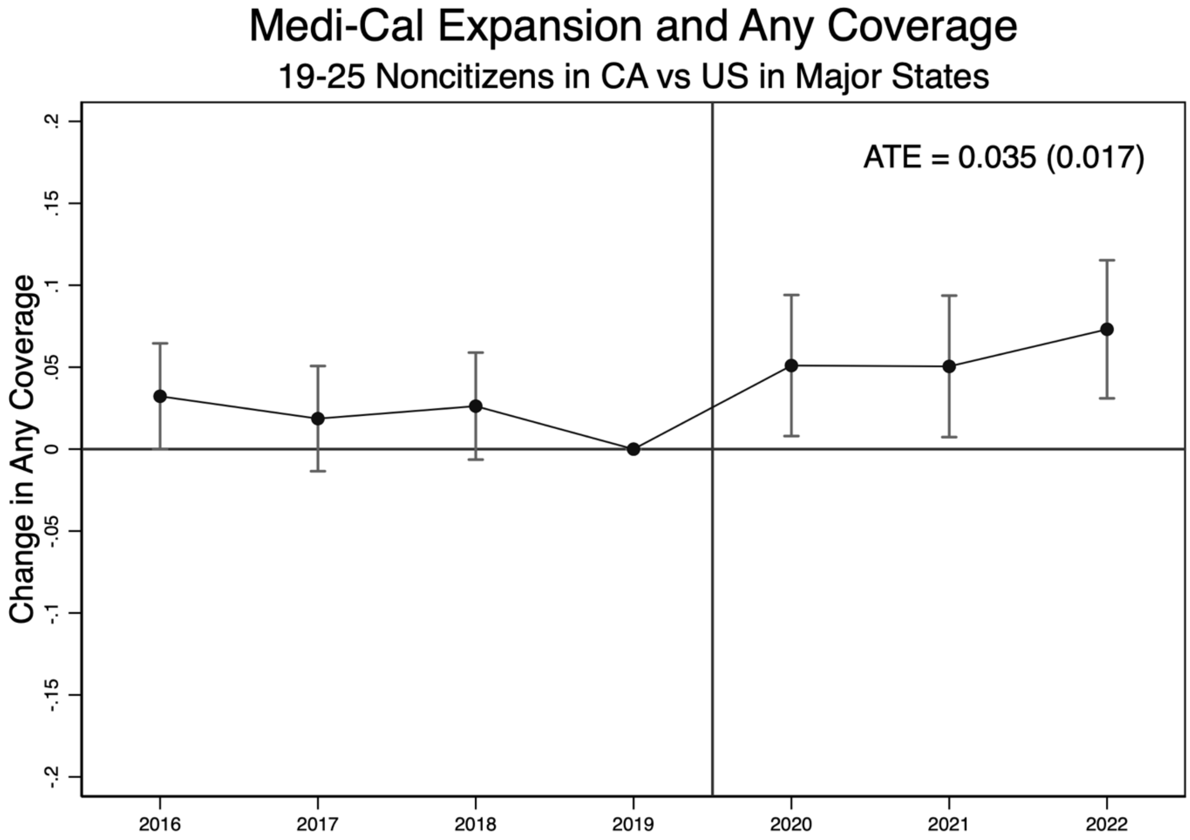


Note: Coefficient estimates and 95% confidence intervals from a triple difference event study are shown. The regression is weighted using ACS sampling weights and strata and features PUMA, year, and age fixed effects, as well as the full vector of demographic controls included in the even-numbered columns of Table 2. Standard errors are clustered at the PUMA level.

© 2026 Leonard R et al. *JAMA Network Open*.

eFigure 2: Event Study Plot of the Effects of California's Expansion to Undocumented 19-25-Year-Olds on Whether an Individual Reports Having Health Insurance Coverage Through Medicaid

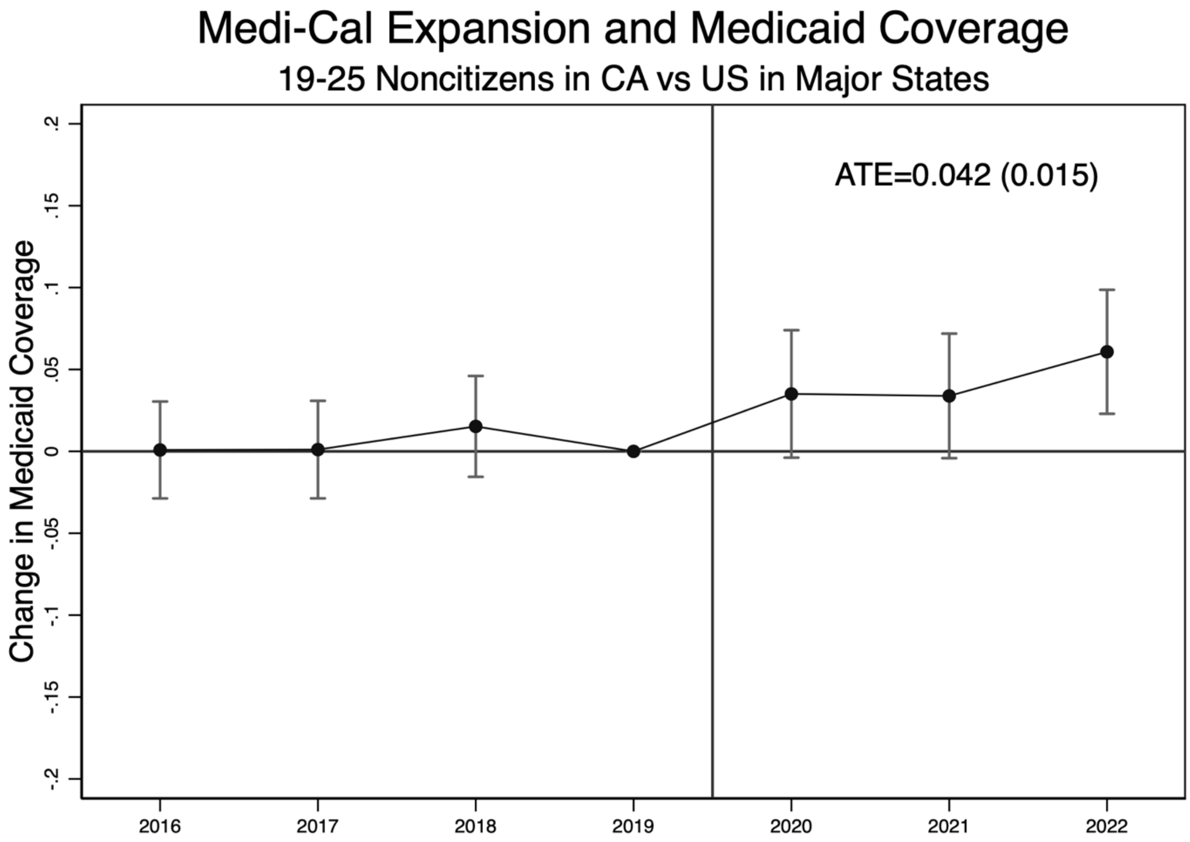


Note: Coefficient estimates and 95% confidence intervals from a triple difference event study are shown. The regression is weighted using ACS sampling weights and strata and features PUMA, year, and age fixed effects, as well as the full vector of demographic controls included in the even-numbered columns of Table 2. Standard errors are clustered at the PUMA level.

© 2026 Leonard R et al. *JAMA Network Open*.

eFigure 3: Event Study Plot of the Effects of California's Expansion to Undocumented 19-25-Year-Olds on Whether an Individual Reports Having Private Health Insurance Coverage

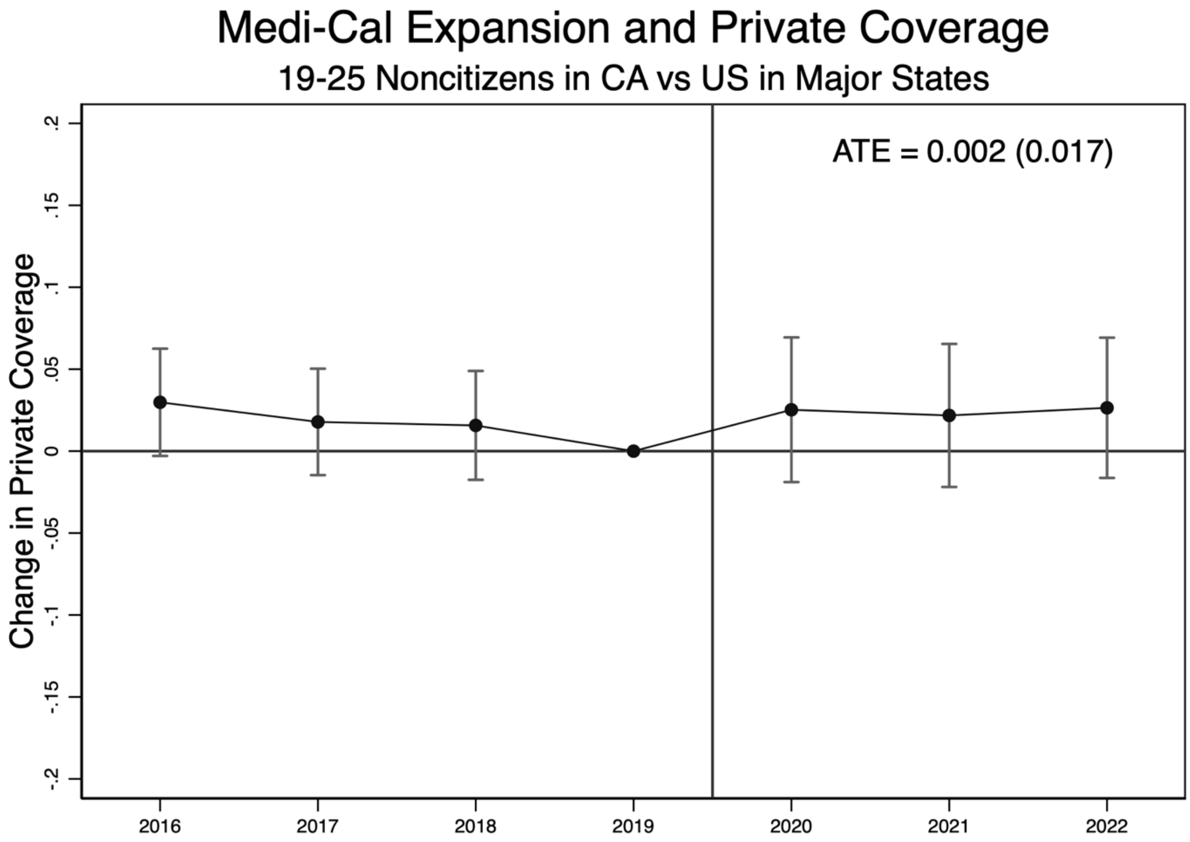


Note: Coefficient estimates and 95% confidence intervals from a triple difference event study are shown. The regression is weighted using ACS sampling weights and strata and features PUMA, year, and age fixed effects, as well as the full vector of demographic controls included in the even-numbered columns of Table 2. Standard errors are clustered at the PUMA level.

© 2026 Leonard R et al. *JAMA Network Open*.

eTable 1. Triple Difference Estimates of the Effects of California's Expansion to Undocumented 19-25-Year-Olds on Main Health Insurance Outcomes, Sensitivity to Inclusion/Exclusion of Potentially Contaminated Data Years

| | Percentage Point Change in Outcome / 100 (SE) | | |
|---|---|---|---|
| | (1)<br>Any Coverage | (2)<br>Medicaid | (3)<br>Private HI |
| **Panel I: With 2020** | | | |
| CA*19-25*post | 0.039* | 0.040** | 0.008 |
| | (0.016) | (0.014) | (0.017) |
| Observations | 138796 | 138796 | 138796 |
| **Panel II: No 2021** | | | |
| CA*19-25*post | 0.043* | 0.045** | 0.009 |
| | (0.018) | (0.015) | (0.018) |
| Observations | 119922 | 119922 | 119922 |
| **Panel III: No 2020 or 2021** | | | |
| CA*19-25*post | 0.040+ | 0.054** | -0.003 |
| | (0.021) | (0.018) | (0.021) |
| Observations | 105162 | 105162 | 105162 |

+ $p < 0.1$, * $p < 0.05$, ** $p < 0.01$, *** $p < 0.001$

Note: All regressions include ACS data for 19-32-year-old noncitizens in states containing at least one county with an estimated undocumented population of at least 100,000. Regressions in Panel I include ACS data from all years 2016-2022. Panel II includes all years except 2021. Panel III contains all years except 2020 and 2021. All regressions are weighted using ACS sampling weights and strata. Standard errors clustered at the PUMA level are in parentheses. All regressions include PUMA, year, and age fixed effects, as well as the full vector of demographic controls included in Table 2.

© 2026 Leonard R et al. *JAMA Network Open*.

eTable 2: Triple Difference Estimates of the Effects of California's Expansion to Undocumented 19-25-Year-Olds on Health Insurance Outcomes Defined by SHADAC and KFF hierarchies

| | Percentage Point Change in Outcome / 100 (SE) | | | | | |
|---|---|---|---|---|---|---|
| | (1) | (2) | (3) | (4) | (5) | (6) |
| Panel I: SHADAC | | | | | | |
| | Medicare | Employer/ Military | Medicaid | Direct Purchase | No Coverage | - |
| CA*19-25*post | -0.003 | -0.000 | 0.037* | 0.001 | -0.035* | - |
| | (0.003) | (0.017) | (0.015) | (0.012) | (0.017) | - |
| Observations | 124036 | 124036 | 124036 | 124036 | 124036 | - |
| | | | | | | |
| Panel II: KFF | | | | | | |
| | Medicaid | Employer | Medicare | Military | Direct Purchase | No Coverage |
| CA*19-25*post | 0.042** | -0.008 | -0.003 | 0.003 | 0.001 | -0.035* |
| | (0.015) | (0.017) | (0.002) | (0.002) | (0.012) | (0.017) |
| Observations | 124036 | 124036 | 124036 | 124036 | 124036 | 124036 |

+ $p < 0.1$, * $p < 0.05$, ** $p < 0.01$, *** $p < 0.001$

Note: All regressions include ACS data for 19-32-year-old noncitizens in states containing at least one county with an estimated undocumented population of at least 100,000. All regressions are run on samples from 2016-2022, excluding 2020. Panel I features results from coding binary health insurance outcomes according to State Health Access Data Assistance Center hierarchy, and Panel II does so using the Kaiser Family Foundation hierarchy. All regressions are weighted using ACS sampling weights and strata. Standard errors clustered at the PUMA level are in parentheses. All regressions include PUMA, year, and age fixed effects, as well as the full vector of demographic controls included in the even-numbered columns of Table 2.

© 2026 Leonard R et al. *JAMA Network Open*.

eTable 3: Triple Difference Estimates of Medi-Cal Expansion on Health Insurance Outcomes, Sensitivity Analysis of Inclusion of Educational Attainment

| | Percentage Point Change in Outcome / 100 (SE) | | |
|---|---|---|---|
| | (1) | (2) | (3) |
| | anycov | hascaid | hasprivate |
| CA*19-25*post | 0.034* | 0.042** | 0.002 |
| | (0.017) | (0.015) | (0.017) |
| Male | -0.057*** | -0.077*** | 0.017*** |
| | (0.003) | (0.003) | (0.003) |
| Hispanic | -0.150*** | 0.010+ | -0.161*** |
| | (0.007) | (0.005) | (0.007) |
| Black | -0.002 | 0.037*** | -0.037*** |
| | (0.011) | (0.008) | (0.011) |
| Asian | 0.054*** | -0.016** | 0.071*** |
| | (0.006) | (0.005) | (0.007) |
| Other race | 0.001 | 0.043*** | -0.044** |
| | (0.013) | (0.011) | (0.014) |
| Married | 0.045*** | 0.021*** | 0.024*** |
| | (0.004) | (0.003) | (0.004) |
| Less than grade 12 | -0.067*** | 0.001 | -0.073*** |
| | (0.012) | (0.010) | (0.010) |
| Completed grade 12 | 0.090*** | -0.010 | 0.097*** |
| | (0.011) | (0.009) | (0.010) |
| 1-2 years college | 0.190*** | -0.040*** | 0.228*** |
| | (0.012) | (0.010) | (0.011) |
| 4 years college | 0.238*** | -0.122*** | 0.355*** |
| | (0.012) | (0.010) | (0.011) |
| 5+ years college | 0.292*** | -0.165*** | 0.450*** |
| | (0.012) | (0.010) | (0.011) |
| Observations | 124036 | 124036 | 124036 |

+ $p < 0.1$, * $p < 0.05$, ** $p < 0.01$, *** $p < 0.001$

Note: All regressions include ACS data for 19-32-year-old noncitizens in states containing at least one county with an estimated undocumented population of at least 100,000, as described in the text. All regressions are run on samples from 2016-2022, excluding 2020, due to data concerns arising from the COVID-19 pandemic. All regressions are weighted using ACS sampling weights. Standard errors clustered at the PUMA level are in parentheses. All regressions include PUMA, year, and age fixed effects, as well as controls as described in the text, and controls for categories of educational attainment.

© 2026 Leonard R et al. *JAMA Network Open*.

eTable 4: Triple Difference Estimates of Medi-Cal Expansion on Health Insurance Outcomes, Sensitivity Analysis of Exclusion of Demographic Controls

| | Percentage Point Change in Outcome / 100 (SE) | | |
|---|---|---|---|
| | (1) | (2) | (3) |
| | anycov | hascaid | hasprivate |
| CA*19-25*post | 0.023 | 0.046** | -0.014 |
| | (0.018) | (0.015) | (0.019) |
| Observations | 124036 | 124036 | 124036 |

+ $p < 0.1$, * $p < 0.05$, ** $p < 0.01$, *** $p < 0.001$

Note: All regressions include ACS data for 19-32-year-old noncitizens in states containing at least one county with an estimated undocumented population of at least 100,000, as described in the text. All regressions are run on samples from 2016-2022, excluding 2020, due to data concerns arising from the COVID-19 pandemic. All regressions are weighted using ACS sampling weights. Standard errors clustered at the PUMA level are in parentheses. All regressions include PUMA, year, and age fixed effects.

© 2026 Leonard R et al. *JAMA Network Open*.

eTable 5: Triple Difference Estimates of the Effects of California's Expansion to Undocumented 19-25-Year-Olds on Main Health Insurance Outcomes, Sensitivity to Restricting Sample to Disadvantaged Economic Subgroups

| | Percentage Point Change in Outcome / 100 (SE) | | |
|---|---|---|---|
| | (1)<br>Any<br>Coverage | (2)<br>Medicaid<br>Coverage | (3)<br>Private<br>Coverage |
| Panel I: No Parents in Household, <=138%FPL | | | |
| CA*19-25*post | 0.048 | 0.042 | 0.009 |
| | (0.035) | (0.031) | (0.032) |
| Observations | 35721 | 35721 | 35721 |
| Panel II: Lowest-Income Counties | | | |
| CA*19-25*post | 0.042 | 0.059 | 0.002 |
| | (0.062) | (0.057) | (0.060) |
| Observations | 11149 | 11149 | 11149 |
| Panel III: Highest-Income Counties | | | |
| CA*19-25*post | 0.035 | 0.038 | 0.019 |
| | (0.034) | (0.030) | (0.037) |
| Observations | 27970 | 27970 | 27970 |

$^{+}$ $p < 0.1$, $^{*}$ $p < 0.05$, $^{**}$ $p < 0.01$, $^{***}$ $p < 0.001$

Note: All regressions include ACS data for 19-32-year-old noncitizens in states containing at least one county with an estimated undocumented population of at least 100,000. Panel I restricts the sample to 19-32-year-old noncitizens who do not live with at least one parent and whose total family income is at/below 138% of the federal poverty line. Panel II restricts the sample to 19-32-year-old noncitizens in counties falling in the top quartile for 19-32-year-old noncitizens living at or below 138% of the federal poverty line. Panel III restricts the sample to 19-32-year-old noncitizens in counties falling in the bottom quartile for 19-32-year-old noncitizens living at or below 138% of the federal poverty line. All regressions are run on samples from 2016-2022, excluding 2020. All regressions are weighted using ACS sampling weights and strata. Standard errors clustered at the PUMA level are in parentheses. All regressions include PUMA, year, and age fixed effects, as well as the full vector of demographic controls included in the even-numbered columns of Table 2.

© 2026 Leonard R et al. *JAMA Network Open*.

eTable 6: Difference-in-Differences Estimates of Medi-Cal Expansion on Health Insurance Outcomes

| | Percentage Point Change in Outcome / 100 (SE) | | | | | |
|---|---|---|---|---|---|---|
| | (1) Any Coverage | (2) Any Coverage | (3) Medicaid | (4) Medicaid | (5) Private HI | (6) Private HI |
| 19-25*post | -0.012 | 0.004 | 0.054*** | 0.043*** | -0.056*** | -0.029* |
| | (0.011) | (0.011) | (0.011) | (0.011) | (0.013) | (0.011) |
| Male | | -0.081*** | | -0.103*** | | 0.021*** |
| | | (0.005) | | (0.004) | | (0.005) |
| Hispanic | | -0.218*** | | 0.110*** | | -0.329*** |
| | | (0.009) | | (0.009) | | (0.011) |
| Black | | -0.051* | | 0.023 | | -0.073** |
| | | (0.021) | | (0.022) | | (0.026) |
| Asian | | 0.027*** | | -0.060*** | | 0.088*** |
| | | (0.008) | | (0.009) | | (0.011) |
| Other race | | -0.040* | | 0.058** | | -0.099*** |
| | | (0.019) | | (0.022) | | (0.024) |
| Married | | 0.054*** | | 0.020*** | | 0.032*** |
| | | (0.006) | | (0.006) | | (0.006) |
| Observations | 52288 | 52288 | 52288 | 52288 | 52288 | 52288 |

$^{+}$ $p < 0.1$, $^{*}$ $p < 0.05$, $^{**}$ $p < 0.01$, $^{***}$ $p < 0.001$

Note: All regressions include ACS data for 19-32-year-old noncitizens in California. All regressions are run on samples from 2016-2022, excluding 2020, due to data concerns arising from the COVID-19 pandemic. All regressions are weighted using ACS sampling weights. Standard errors clustered at the PUMA level are in parentheses. All regressions include PUMA, year, and age fixed effects.

© 2026 Leonard R et al. *JAMA Network Open*.
