# Supplementary material for: The California 2020 Medi-Cal Expansion to Young Adults and Coverage Among Noncitizens
Source: JAMA Netw Open. 2026 May 13;9(5):e2612332. doi: 10.1001/jamanetworkopen.2026.12332 (PMC13173380; doi:10.1001/jamanetworkopen.2026.12332)
Supplement: Supplement 2. — Data Sharing Statement [file jamanetwopen-e2612332-s002.pdf]

Data Sharing Statement

Leonard. The California 2020 Medi-Cal Expansion to Young Adults and Coverage Among Noncitizens. *JAMA Netw Open*. Published May 13, 2026.
doi:10.1001/jamanetworkopen.2026.12332

Data
Data available: No

Additional Information
Explanation for why data not available: Data for this study are publicly available here: https://usa.ipums.org/usa/
